# Lipidomic Profiling of Edible Japanese Sea Urchins by LC–MS

**DOI:** 10.3390/foods14132268

**Published:** 2025-06-26

**Authors:** Sahana Amai, Kisara Yuki, Siddabasave Gowda B. Gowda, Divyavani Gowda, Shu-Ping Hui

**Affiliations:** 1 Graduate School of Global Food Resources, Hokkaido University, Kita-9, Nishi-9, Kita-Ku, Sapporo 060-0809, Japan; sahanaa158@gmail.com; 2 Faculty of Health Sciences, Hokkaido University, Kita-12, Nishi-5, Kita-Ku, Sapporo 060-0812, Japan; yuuki.kisara.q7@elms.hokudai.ac.jp (K.Y.); divyavani@hs.hokudai.ac.jp (D.G.)

**Keywords:** sea urchin, liquid chromatography, mass spectrometry, lipidomics

## Abstract

Sea urchins (*Echinoidea*) are marine echinoderms commonly consumed as seafood in East Asia. To date, various metabolic components of sea urchins have been analyzed, and their health benefits for humans have also been attracting attention. Lipids are the major biomolecules present in sea urchins. However, the comprehensive lipid profiling of sea urchins is limited. In this study, we aimed to perform the comprehensive lipid profiling of six types of sea urchins using liquid chromatography–mass spectrometry (LC/MS). The application of untargeted lipidomics led to the identification of 281 lipid molecular species in six varieties of fresh sea urchin gonads. Each lipid metabolite was identified based on its retention time and MS/MS fragmentation pattern. The results of the analysis showed the highest abundance of lipid percentage in Kitamurasakiuni (14.3%), followed by Hokuyobafununi (12.4%). In all the analyzed sea urchins, glycerolipids such as triacylglycerols were found to be the most abundant lipid components. Multivariate analysis revealed that Murasakiuni showed a different lipid profile from the other types. Interestingly, the polyunsaturated fatty acid to saturated fatty acid ratios and health-related nutritional indices factors were found to be higher in Hokuyobafununi compared to other varieties. The ω-3 fatty acids, such as docosapentaenoic acid (FA 22:6) and eicosapentaenoic acid (FA 20:5), were also abundant in Hokuyobafununi. Lipids such as ether and N-acyl-type lysophosphatidylethanolamines were detected for the first time in sea urchins. This study highlights the nutritional significance of sea urchins and their potential use in the development of functional foods.

## 1. Introduction

Globally, the demand for nutritious food is very high due to the increasing population, and seafood is considered a good dietary source as it has a positive impact on health because of the presence of unsaturated fatty acids [1,2]. Sea urchins are marine animals belonging to the echinoderm family. Approximately 20 species of sea urchins are consumed across the globe, and 6 species are used as dietary foods in Japan [3]. Gonads are considered the edible part of sea urchins due to their unique flavor and taste [4]. The sea urchin industry has important economic value all over the world, as gonads are in high demand [5]. The sea urchin industry is considered significant, with Japan consuming 80–90% of the global supply (50,000–60,000 tons annually). In Sardinia, an island belonging to Italy, sea urchins are consumed as a traditional food, with an annual per capita consumption of about 1.1 kg. During the off-season, from spring to autumn, domestic production in Japan cannot meet demand, leading to imports from countries such as Russia, Chile, Canada, the United States, and others [6]. Metabolites, such as lipids, carbohydrates, and carotenoids, influence the quality of the gonads [7,8]. Several sea urchin-derived metabolites, such as polysaccharides, are shown to have various anti-inflammatory and anti-oxidant properties, such that they can be utilized as a healthy, functional food source [9,10,11].

Lipids are organic compounds that play an important role in providing the structural basis for the cell membrane and an energy source for the organism [12]. These molecules are of different types, such as fatty acids, phospholipids, sterols, and sphingolipids [13,14]. As some PUFAs, such as linoleic acid and linolenic acid, are not synthesized in the human body, they serve as the key component of a healthy diet [15]. The high nutritional value of the sea urchin gonad is due to the presence of polyunsaturated fatty acids (PUFAs) and eicosapentaenoic fatty acid (EPA), which are important for maintaining good health [16]. Also, the intake of fish and shellfish, which are rich sources of n-3 PUFAs, can help lower the risk of heart diseases, diabetes, and immune-related conditions, providing various health benefits [17]. Therefore, PUFAs can be considered a potential target for the treatment of cardiovascular diseases, inflammation, cancer, and hypertension [18]. A study by Kalogeropoulos et al. [19] reported that the eggs of the sea urchins are enriched with EPAs, long-chain ω-3 polyunsaturated fatty acids, making it a healthy dietary source, which can provide up to 234.1 mg of the ω-3 fatty acid per 100 g of consumption. Previously, the nutrient composition of the sea urchin gonad was analyzed by Archana et al. and was found to have an abundant concentration of PUFAs and amino acids, causing a significant effect on cardiovascular health and serving as a potential source for the nutraceutical industry [20]. Meanwhile, a lipidomic study by Wang et al. discovered that the ether-phospholipid component in the sea urchin was involved in the regulation of cholesterol levels in serum and served as the nutraceutical for atherosclerotic dyslipidemia [21].

Moreover, analytical techniques such as liquid chromatography–tandem mass spectrometry (LC–MS/MS), thin-layer chromatography–flame ionization detection (TLC–FID), and high-performance liquid chromatography–mass spectrometry (HPLC–MS/MS) have been used to study the lipidomic and lipid composition of the sea urchins [22]. In fact, we have previously applied an untargeted LC–MS technique to characterize the seafood lipidome [23,24]. A recent study by Zhou et al. showed that partial lipid composition analysis, such as glycerophospholipids (GPs), was characterized in the gonads of three species of sea urchins [25]. However, there are limited reports on the comprehensive analysis of lipid nutrient levels in sea urchin gonads native to Japan. Therefore, in this study, we aimed to perform the comprehensive lipid profiling of six different edible sea urchin species using an untargeted liquid chromatography–mass spectrometry (LC–MS) approach, providing detailed information on the various lipid classes present. This analysis paves the way for investigating the potential health benefits of lipids found in sea urchin gonads.

## 2. Materials and Methods

### 2.1. Materials

LC/MS-grade isopropanol, chloroform, and methanol were obtained from Wako Pure Chemical Industries, Ltd. (Osaka, Japan). Ammonium acetate (1 M) was purchased from Sigma-Aldrich (St. Louis, MO, USA). EquiSPLASH lipidomix (1 μg/mL) and oleic acid-d9 (10 μg/mL) were used as internal standards for semi-quantitative analysis. Both the standards were purchased from Avanti Polar Lipids, Inc. (Alabaster, AL, USA).

### 2.2. Sea Urchin Samples

**Figure 1:**
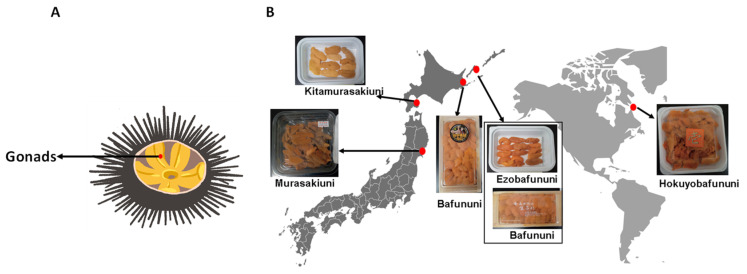
(**A**). An illustration of a sea urchin with exposed gonads. (**B**). Types of sea urchin gonad samples and their collection location.

The gonads of two types of sea urchins, Ezobafununi (*Strongylocentrotus intermedius*, from the four northern islands, Shikotan, Russia) and Kitamurasakiuni (*Strongylocentrotus nudus*, from Hakodate, Hokkaido, Japan), were purchased from Murakami Shoten, a company located in Hokuto, Hokkaido, Japan. Four other types, including Hokuyobafununi (*Strongylocentrotus droebachiensis*, from Canada), Bafununi (*Hemicentrotus pulcherrimus*, from the four northern islands, Russia), farmed Bafununi (*Hemicentrotus pulcherrimus*, from Hamanaka, Hokkaido, Japan), and Murasakiuni (*Heliocidaris crassispina*, from Matsushima, Miyagi, Japan), were purchased from Yoshizen, a company located in Toyosu, Tokyo, Japan. All the samples and their collected locations are shown in Figure 1. The gonads of Ezobafununi, Bafununi, and Hokuyubafununi were female, whereas Kitamurasakiuni and Murasakiuni were male. Harvesting seasons for sea urchins vary from country to country, but in Japan, summer is the most common season to harvest sea urchins. However, for our study, the samples were collected in winter (February 2024). All the collected samples were fresh and stored at −80 °C until the analysis.

### 2.3. Lipid Extraction

The lipid extraction of sea urchin gonads was performed by the modified Folch extraction method [24,26]. About 150 mg of sea urchin gonads was placed in a 2.0 mL Eppendorf tube, and 5–6 ceramic beads were added and then homogenized for 30 s at room temperature. After that, 1.5 mL of methanol was added and homogenized for an additional 30 s. About 200 μL of the homogenate was transferred to a new 2.0 mL Eppendorf tube, and 200 μL of the internal standard mixture (EquiSPLASH lipidomix (1 μg/mL) and oleic acid-d9 (10 μg/mL) in methanol) were added. Then, the samples were vortexed at 3500 rpm for 5 min at room temperature. After that, 800 μL of chloroform and 200 μL of Milli-Q were added to the tube, vortexed for 5 min, and centrifuged at 3500 rpm for 5 min. The lower chloroform layer was transferred to a new vial. The extraction process was repeated by adding 800 μL of chloroform to the aqueous phase. The combined chloroform extracts were dried using an EYELA CVE 3000 centrifugal evaporator (Rikakikai Co., Ltd., Tokyo, Japan), under vacuum at 4 °C for 3 h. Then, the residue was redissolved in 100 μL of methanol, vortexed for 1 min, and centrifuged at 15,000 rpm, at 4 °C, for 10 min. The centrifugate was transferred to an LC-MS vial for analysis.

### 2.4. LC/MS Analysis

The LC system (Shimadzu Corporation, Kyoto, Japan) and LTQ Orbitrap mass spectrometer (Thermo Fisher Scientific Inc., San Jose, CA, USA) were used for the analysis in both negative and positive ionization modes. An Atlantis T3 C18 column (2.1 × 150 mm, 3 mm, Water, Milford, MA, USA) maintained at an oven temperature of 40 °C was used for the chromatographic separation of lipids. The mobile phases used were (A) 10 mM aqueous ammonium acetate, (B) isopropanol, and (C) methanol. The sample injection volume was 10 μL, and the parameters considered for mass spectrometry analysis were the same as previously reported [27]. The data obtained from MS were processed for alignment, peak extraction, and the identification of lipid molecules using MS-DIAL software ver. 4.9. The peak area integration was performed by Xcalibur ver. 4.6. Each lipid was quantified by selecting a suitable internal standard and calculating the ratio of their peak areas according to the Lipid Standard Initiative guidelines, quantification level 3 (https://lipidomicstandards.org/, accessed on 15 April 2024). Statistical analysis and data visualization were performed by MetaboAnalyst ver. 6.0 and GraphPad Prism ver. 8 software.

### 2.5. Determination of Total Lipid Percentage

The gonads of the sea urchins were weighed (approximately 500 mg) and placed in a 15 mL centrifuge tube. About 6 mL of a mixture of chloroform and methanol (chloroform/methanol = 2:1 *v*/*v*) and 1 mL of Milli-Q water were added to the tube. The samples were vortexed at 3500 rpm for 5 min and centrifuged at 3500 rpm at 4 °C for 10 min. After that, a new 15 mL tube was weighed, and the layer of chloroform was transferred onto it. Furthermore, 6 mL of the chloroform and methanol mixture (2:1 *v*/*v*) was added to the residue, vortexed for 1 min, centrifuged at 3500 rpm at 4 °C for 10 min, and the chloroform layer was collected into the same tube. The combined chloroform extract was dried at 4 °C using a centrifuge evaporator under vacuum for 3 h. The tubes containing the residue were then weighed. The weight of the total lipids was calculated by subtracting the weight of the empty tube from that of the tube containing lipid residue. Triplicate measurements were made for each type of sea urchin.

### 2.6. Calculation of Nutritional Indices of Sea Urchins

Nutritional indices such as the index of atherogenicity (IA), the health promotion index (HPI), hypo/hypercholesterolemic ratio (HH), and lipid quality (LQ) were calculated using the following equations to evaluate the nutritional value of sea urchins [28].

IA = [C12:0 + (4 × C14:0) + C16:0]/ΣUFA

HPI = ΣUFA/[C12:0 + (4 × C14:0) + C16:0]

HH = (C18:1 + ΣPUFA)/(C12:0 + C14:0 + 16:0)

LQ = 100 × (C22:6 + C20:5)/ΣFA

## 3. Results and Discussion

### 3.1. The Distribution of the Amount of Lipids in Sea Urchins Based on Lipid Classes

**Figure 2:**
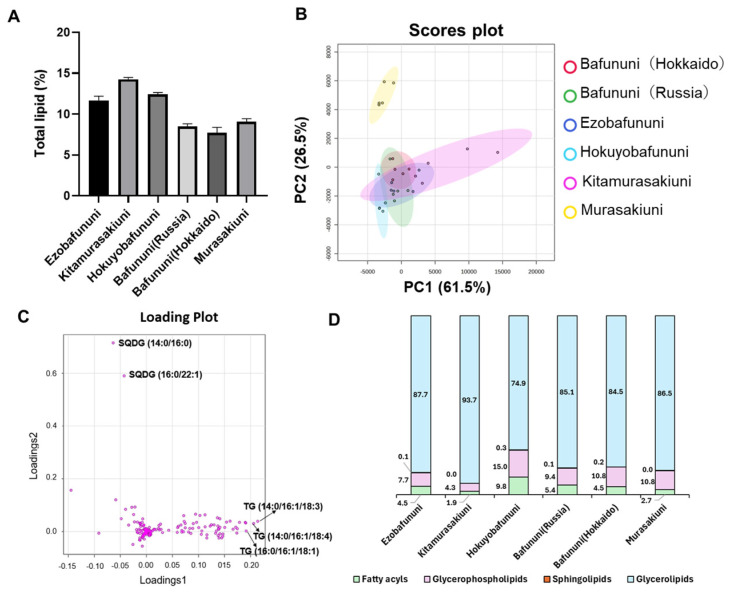
(**A**). Percentage of total lipids per fresh weight determined in sea urchins. (**B**). Principal component analysis (PCA) scores plot of lipid species from six species of sea urchin. (**C**). Loading plots of lipid species from six species of sea urchins where TGs are the major contributor to group separation (**D**). Percentage distribution of four lipid classes among six types of sea urchins (sphingolipids, indicated by orange color, are difficult to see in figure due to their very low abundance of <1%).

An untargeted lipidomic study of the sea urchin gonad was conducted in both positive and negative modes of ionization. The list of all the identified lipid species and their relative concentrations in all sea urchin samples is provided in the Supporting Information (Table S1). A total of 281 lipid molecular species were identified. These were identified based on MS and MS/MS fragmentation spectra and by comparing experimental and computational spectra using MS-DIAL. Relative quantification was achieved by multiplying the peak area ratios of annotated lipids to the internal standard by the known concentration of the added internal standard. Figure 2A shows the percentage of total lipid in six types of sea urchins. The highest percentage composition was observed in Kitamurasakiuni (14.3% FW), followed by Hokuyobafununi (12.4% FW). The partial least squares discriminant analysis score plots exhibited a distinctive and evident group separation of Murasakiuni, followed by Kitamurasakiuni, from the other four species of sea urchins, as shown in Figure 2B. Component 1 showed a partial variance of 61.5%, which is more than component 2, contributing 26.5% towards the total variance. The lipid composition is affected by the higher positive and negative values of the loading scores for lipid species shown in Figure 2C. The unique group separation observed was due to lipids such as sulfoquinovosyl diacylglycerol (SQDG) (14:0/16:0) and SQDG (16:0/22:1) with negative loading scores, while triacylglycerol (TG) (14:0/16:1/18:3), TG (14:0/16:1/18:4), and TG (16:1/16:1/18:1) had positive loading scores. A glycerolipid, SQDG, is reported to show antitumor [29] and antiviral [30] properties and has inhibitory activity against the DNA polymerase enzyme in mammals [31]. Figure 2D depicts the distribution of the relative abundance of major classes of lipids across six different species of sea urchins according to LIPID MAP guidelines. It shows that the relative amount of glycerolipid is higher in Kitamurasakiuni. By contrast, fatty acyls are predominant in the case of Hokuyobafununi. Also, the relative concentration of the sphingolipids is negligible in all the species of sea urchins, at <1%.

### 3.2. Hierarchical Cluster Correlation Analysis and Composition of Free Fatty Acid

**Figure 3:**
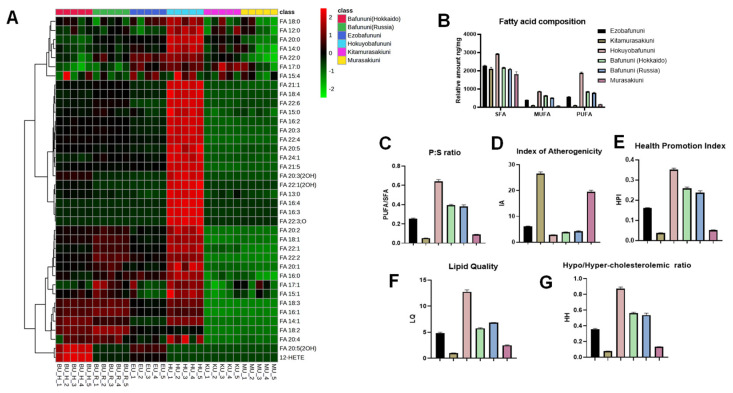
(**A**). Heatmap of free fatty acids showing concentration variations. (**B**). Fatty acid composition based on unsaturation in six types of sea urchins. (**C**). PUFA to SFA (P:S) ratio in sea urchins (**D**). Index of atherogenicity [IA]. (**E**). Health promotion index [HPI]. (**F**). Lipid quality [LQ]. (**G**). Hypo/hyper-cholesteroleimic ratio [HH ratio].

The untargeted LC-MS analysis of six varieties of sea urchin gonads identified the presence of 37 free fatty acids. These fatty acids are classified into three types based on their unsaturation, including saturated fatty acids (SFAs), mono-unsaturated fatty acids (MUFAs), and polyunsaturated fatty acids (PUFAs) [32]. The heatmap visualization shown in Figure 3A explains the relative concentration of the free fatty acids in each sea urchin variety. The intense red color in the heatmap indicates the highest concentration, while the intense blue color indicates the lowest concentration of the free fatty acid. The obtained results demonstrate that the concentration of each fatty acid tends to be higher in Hokuyobafununi, followed by the relative amount of FA20:5 (OH), with FA15:4 being predominant in Bafununi (Hokkaido). Also, the fatty acids FA20:5 (EPA) and FA22:6 (DHA) are both abundant in Hokuyobafununi. Both EPA and DHA are known to act as anti-inflammatory lipids and help improve brain and heart functions [33]. Various studies have been carried out to understand the impact that the intake of food enriched with EPA and DHA has on plasma lipids [34,35,36]. These n-3 PUFAs have been shown to play a positive role in regulating lipid metabolism and reducing the risk of cardiovascular diseases and auto-immune disorders [37]. As observed from Figure 3B, the Hokuyobafununi species exhibited a relatively higher amount of SFA and PUFA compared to Kitamurasakiuni, exhibiting the lowest amount of PUFA and MUFA. However, Murasakiuni also showed the lowest concentration of SFA. Further, the ratio of PUFA to SFA (P:S) is shown in Figure 3C. Our results show that Hokuyobafununi has the highest P:S ratio, followed by Bafununi (Russia), and the lowest ratio belongs to Kitamurasakiuni species. As reported earlier by Kang et al., cardiovascular diseases can be prevented by increasing the dietary P:S ratio [38].

### 3.3. Analysis of Nutritional Indices of Sea Urchin Samples

The nutritional indices of the lipids of six species of sea urchins are depicted in Figure 3D. Kitamurasakiuni exhibited a higher index of atherogenicity (IA) value, followed by Murasakiuni, and a lower index in Hokuyobafununi compared to other species. It was found that an atherogenic index can be considered a risk factor for cardiovascular disease. Also, the higher the value of IA, the greater the risk of coronary artery disease (CAD) [39]. In the previous report by Ozbey et al., the health promotion index (HPI) value calculated for the dairy product was calculated and found to be high. The increase in the value of HPI had a positive impact on human health [40]. Among the characterized species, Hokuyobafununi was found to have the maximum HPI value, indicating the probability of benefits for human health compared to the Kitamurasakiuni species, which has a minimum HPI value, as shown in Figure 3E. Similarly, the lipid quality of the Hokuyobafununi species was very high, while the lipid quality of the Kitamurasakiuni species was the lowest, as shown in Figure 3F. Fish lipid quality (FLQ) is a commonly used method to determine the lipid quality index of marine products; thus, the lipid quality of six species of sea urchin was analyzed using the same method. According to a report by Chen et al., the FLQ value ranges between 13.01 and 36.37 [28]. In a previous study by Gowda et al., the lipidomic profiling of different species of fish was carried out, and the FLQ values of three species, saffron cod, Pacific cod, and shishamo smelt, were found to be (23.51 ± 0.65), (22.45 ± 0.34), and (21.95 ± 0.79), respectively [24]. These values are higher than the FLQ of the Hokuyobafununi species in our study, indicating the better lipid quality of fishes over sea urchin species. The hypo/hyper-cholesteroleimic (HH) ratio can monitor the effect of fatty acids on cardiovascular disease. Compared to the P:S ratio, the value of the HH ratio is considered to provide accurate information regarding the effect of the fatty acid composition on the CAD [24,28]. The HH value was found to be predominant in Hokuyobafununi, whereas it was minimal in the case of Kitamurasakiuni, as shown in Figure 3G.

### 3.4. Hierarchical Correlation Analysis of Complex Lipids Analyzed in Sea Urchins

**Figure 4:**
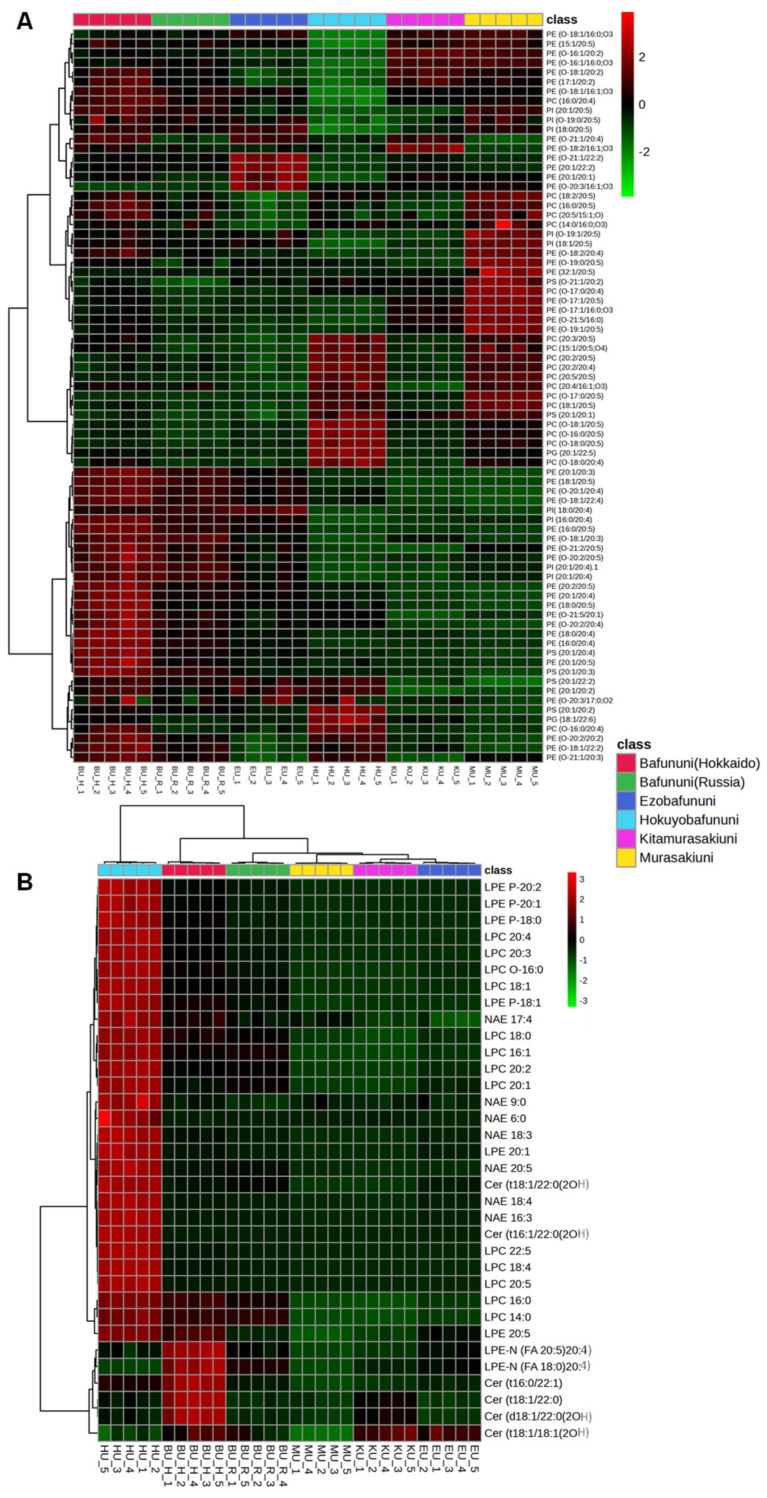
Hierarchical cluster correlation analysis of glycerophospholipids detected in sea urchins. (**A**). Phosphatidylethanolamine (PE), phosphatidylcholine (PC), and phosphatidylinositol (PI). (**B**). Lysophospholipids (LPE), lysophophatidylcholine (LPC), N-acyl-lysophosphatidylethanolamine (LPE-N), N-acyl ethanolamines (NAEs), and ceramides (Cer).

Seafood comprises proteins, lipids, and essential micronutrients, making it a good source of high nutrition. Lipids are vital components of cell membranes and play crucial roles in various physiological functions, including growth, reproduction, immune responses, and energy storage [41]. The relative concentration of complex lipids, such as glycerophospholipids and sphingolipids, analyzed in six species of sea urchins is illustrated by hierarchical correlation heatmaps. Glycerophospholipids such as phosphatidylcholines (PCs), phosphatidylserine (PS), and phosphatidylethanolamine (PE) are identified as having the highest abundance in the case of Bafununi (Hokkaido) compared to other species of sea urchin, as depicted in Figure 4A. Further concentrations of lipids, such as lysophospholipids, NAEs, and ceramides, are also shown in the heatmap, as illustrated in Figure 4B. The abundances of LPE, LPC, NAE, Cer(t18:1/22:0(2OH) and Cer(t16:1/22:0(2OH) were higher in Hokuyobafununi, while the concentrations of LPE-N (FA20:5)20:4, LPE-N (FA18:0)20:4, and ceramide were found to be higher in Bafununi (Hokkaido). Fatty acids are essential components of the human diet and contribute significantly to a wide range of metabolic processes [42]. These complex lipids are also composed of FA 18:3, FA 20:4, FA 20:5, and FA 22:4, which could serve as good sources of PUFAs in seafood. It has been shown that the dietary intake of PUFAs can reduce cholesterol levels in the plasma and thus reduce cardiovascular risks. Furthermore, n-3 and n-6 PUFAs such as EPA and AA are found to play a major role as anti-cancerous agents by reducing the risk of various cancers such as leukemia and breast cancer [43,44]. A study by Zhang et al. indicated that a reduced level of LPCs can lead to a higher risk of insulin resistance and obesity in humans [45]. In our study, Hokuyobafununi can be considered a good source of LPC in the nutritional diet. Ceramides contribute as a major skin component and maintain water permeability [46]. Additionally, the cellular level of ceramide plays an important role in programmed cell death [47]. In a previous study, comparative lipidomics was performed for the sea urchin *Strongylocentrotus intermedius*. The results showed that complex lipids enriched with PUFAs such as FA 20:4 and FA 20:5 could be identified, indicating a high abundance of PUFAs in this species [4].

**Figure 5:**
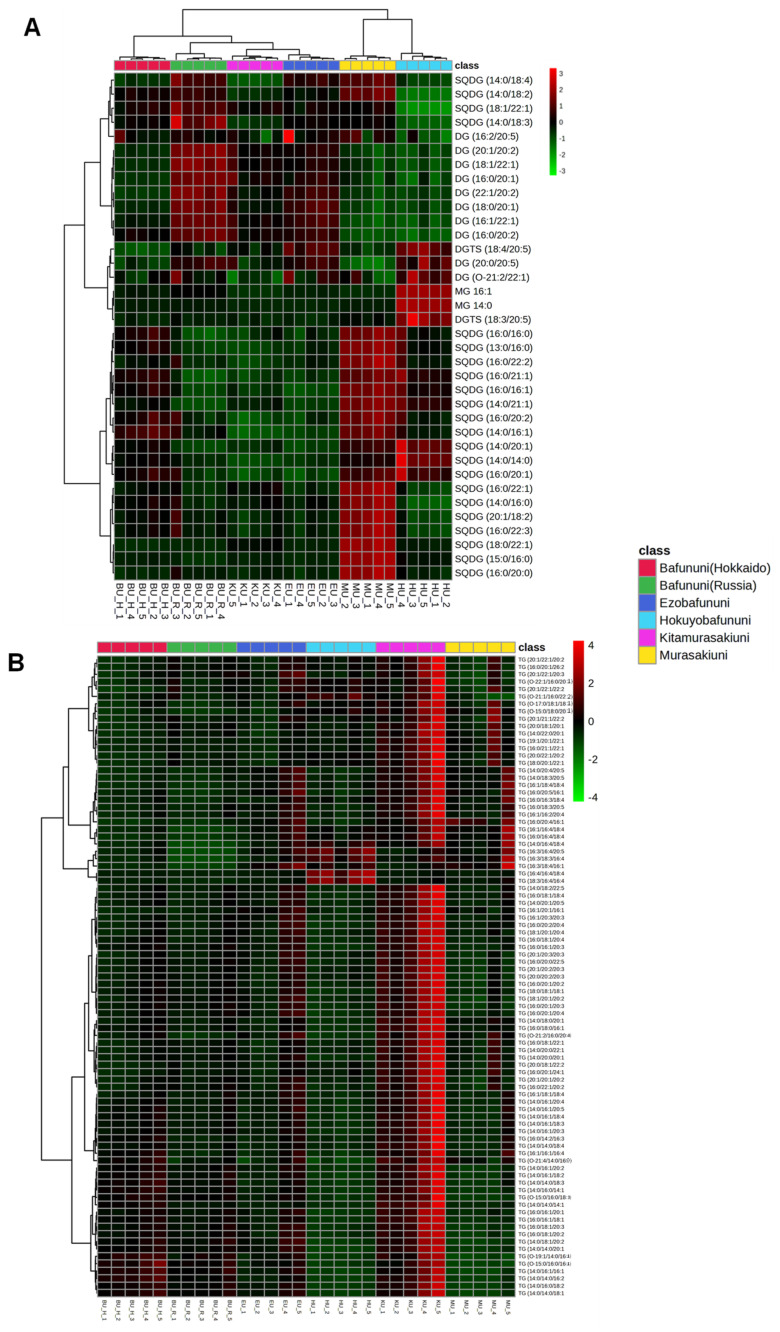
Hierarchical cluster correlation analysis of glycerolipids detected in sea urchins. (**A**). Sulfoquinovosyl diacylglycerol (SQDG), monoacylglycerol (MG), and diacylglycerol (DG). (**B**). Triacylglycerol (TG).

Glycerolipids, such as SQDG, DGTS, MG, and DG, showed higher abundance in Murasakiuni and the lowest concentration in Bafununi (Hokkaido), as shown in Figure 5A. Kitamurasakiuni exhibited the highest abundance of TGs compared to other species used in the analysis, as shown in Figure 5B. Triacylglycerols are stored in the adipose tissue and play an important role in energy storage. The regulation of the metabolism of triacylglycerols can help prevent metabolic diseases such as fatty liver disease, obesity, atherosclerosis, and diabetes [48]. In a recent study, marine organisms were found to be one of the most abundant sources of non-polar lipids [12]. Our analysis results show that TGs are the most predominant non-polar lipid components of sea urchins. Another study involving the dietary intake of DGs (a precursor of TGs) showed decreased plasma lipid levels and reduced the risk of cardiovascular disease [49]. In a previous study, the analysis of the lipid composition of the sea urchin gonads of the *Strongylocentrotidae* family showed that the phospholipids and TGs are the major lipid components, and these results are consistent with our study [50]. As for our knowledge, there are limited studies on the comprehensive lipidomic analysis of sea urchins, and this is the first time we have explored the lipidome of dietary sea urchins consumed in Japan. This study has limitations, such as the analysis results being semiquantitative. Furthermore, environmental factors, harvesting season, and diet may influence the quality of the lipids, which was not considered in this study.

## 4. Conclusions

To conclude, the lipidomic analysis of six different types of sea urchin was conducted using untargeted LC/MS, resulting in the identification of 286 distinct molecular species of lipids. Principal component analysis revealed significant differences in lipid composition between Murasakiuni and Kitamurasakiuni compared to other sea urchin species, highlighting their unique lipid profiles. Additionally, Hokuyobafununi exhibited the highest values of both P:S and HPI, suggesting that this species may offer health benefits and could be a great choice to consider in a healthy diet. Moreover, the heat map visualization of a variety of lipid classes in sea urchins uncovered the fact that TGs are the most abundant lipid subclass available in different types of sea urchins compared to other lipid subclasses. Interestingly, glycerophospholipids and ceramides were found to be more abundant in Bafununi (Hokkaido), whereas Murasakiuni and Kitamurasakiuni exhibited higher concentrations of glycerolipids and TGs, respectively. This detailed lipid profiling indicates that sea urchin lipids could have potential uses in human nutrition and health, especially to enhance diet quality and support overall well-being.

## Data Availability

The raw data supporting the conclusions of this article will be made available by the authors on request.

## Supplementary Materials

The following supporting information can be downloaded at https://www.mdpi.com/article/10.3390/foods14132268/s1. Table S1: Relative concentrations (in µg/g) of lipid species detected in six samples of sea urchin gonads.

## Abbreviations

The following abbreviations are used in this manuscript:

AA	arachidonic acid
CAD	coronary artery disease
Cer	ceramide
DG	diacylglycerol
DGTS	diacylglyceryl trimethylhomoserine
DHA	docosahexaenoic acid
EPA	eicosapentaenoic fatty acid
FA	fatty acid
FLQ	fish lipid quality
FW	fresh weight
HH ratio	hypo/hypercholesterolemic ratio
HPI	health promotion index
IA	index of atherogenicity
LC-MS	liquid chromatography–mass spectrometry
LPC	lysophophatidylcholine
LPE	lysophosphatidylethanolamine
LPE-N	n-acyl-lysophosphatidylethanolamine
LQ	lipid quality
MG	monoacylglycerol
MUFA	mono-unsaturated fatty acid
NAEs	n-acyl ethanolamines
PC	phosphatidylcholines
PE	phosphatidylethanolamine
PS	phosphatidylserine
PUFA	polyunsaturated fatty acid
SFA	saturated fatty acid
SQDG	sulfoquinovosyl diacylglycerol
TG	triacylglycerol
UFA	unsaturated fatty acid
